# Value-Based Health Care: Long-Term Care Insurance for Out-of-Pocket Medical Expenses and Self-Rated Health

**DOI:** 10.3390/ijerph20010192

**Published:** 2022-12-23

**Authors:** Guangbo Ma, Kun Xu

**Affiliations:** Institute of Finance and Public Management, Anhui University of Finance and Economics, Bengbu 233030, China

**Keywords:** long-term care insurance, out-of-pocket medical expenses, self-rated health, value-based health care, differences-in-difference method, propensity score matching

## Abstract

Long-term care insurance (LTCI) is a significant approach in the effort to actively manage aging and the currently unmet need for aged care in China. Based on data from the 2011, 2013, 2015, and 2018 phases of the China Health and Retirement Longitudinal Study, we used the propensity score matching-difference in difference (PSM-DID) approach to explore the impact of LTCI on out-of-pocket medical expenses and self-rated health. Results showed that LTCI can significantly reduce out-of-pocket medical expenses by 37.16% (*p* < 0.01) per year and improve self-rated health by 5.73% (*p* < 0.01), which conforms to the spirit of “value-based health care”. The results were found to be stable in the robustness tests conducted. Currently, China is at the intersection of “low-value-based health care” and “value-based health care”. Improving the health level of aged individuals while keeping medical costs under reasonable control is crucial for formulating and implementing a new round of healthcare reform in China.

## 1. Introduction

China is becoming an increasingly aging society [1]. The number of disabled and semi-disabled individuals is also rising, and health awareness is increasing [2]. These factors have caused a dramatic increase in the demand for healthcare services, endangering the long-term viability of China’s healthcare security system. According to data from China’s seventh census, more than 42 million elderly persons were disabled or semi-disabled as of 2021, making up 16.6% of the entire population, meaning that one in six seniors are unable to care for themselves [3]. It is predicted that by 2050, each working-age person in China will need to support nearly 0.44 older people [4], and this has led to a sharp rise in medical expenses. For example, the average annual growth rate of China’s total health costs was 13.25% between 2010 and 2021, far outpacing the China’s gross domestic product growth rate during the same period. China’s healthcare costs have already soared due to the COVID-19 pandemic, which began in 2020 [5], and for families, the development of home healthcare services is slow [6,7] due to a lack of sufficient time and expertise. Thus, the care needs of the disabled and semi-disabled population have remained unmet for a long time.

To actively respond to the pressure of care needs caused by an aging population [8,9], China introduced a long-term care insurance (LTCI) policy in 2016, beginning with a pilot in 15 cities, which was gradually expanded. There are two essential features of LTCI. First, LTCI funding relies heavily on financial support from social health insurance [10]. According to the National Bureau of Medical Security of China, in 2018, about 80% of LTCI funding came from social medical insurance [11]. Second, healthcare services and supply methods differ. There are four main approaches: (i) providing institutional care only (e.g., Changchun); (ii) providing both institutional care and community care (e.g., Chongqing and Guangzhou); (iii) combining medical care, elderly care, and community home care (e.g., Chengde, Suzhou, Nantong, Shanghai, Qiqihar, Ningbo, and Jingmen); (iv) encouraging home care and providing cash subsidies for informal caregivers (e.g., Qingdao, Chengdu, Shihezi, Shangrao, and Anqing).

However, the jury is still out as to whether LTCI affects medical expenses and health. On the one hand, LTCI reduces medical expenses. It can effectively reduce bed blocking and delayed discharge [12], thus resulting in a “substitution effect”. Meanwhile, the financial subsidies provided by LTCI have the potential to improve the nutrition and other health inputs of beneficiaries [13,14], thus leading to a decrease in the utilization rate of medical services and medical costs. This is consistent with value-based health care [15]. On the other hand, LTCI can increase the total medical expenses while reducing individual costs [16]. This is because LTCI uses a third-party payment mechanism, which primarily reduces the cost of the actual expenditure of an individual or family. Individuals who follow the laws of supply and demand will require more healthcare services as a result of receiving them at lower prices. Therefore, the current study examined whether LTCI can influence out-of-pocket medical expenses and health and whether this is in line with value-based health care.

There are two difficulties in exploring the above issues. First, there are many instances of missing data in the existing micro household survey, and there are differences in the criteria used to measure indicators, leading to low credibility. Meanwhile, effectively dealing with the endogeneity problem poses a significant challenge. Therefore, we selected the PSM-DID method to empirically test the impact of LTCI on out-of-pocket medical expenses and self-rated health and better address the selectivity bias problem by using a series of measures (e.g., parallel trend test, placebo effect, and changing the PSM matching mode) in the robustness testing.

**Figure 1:**
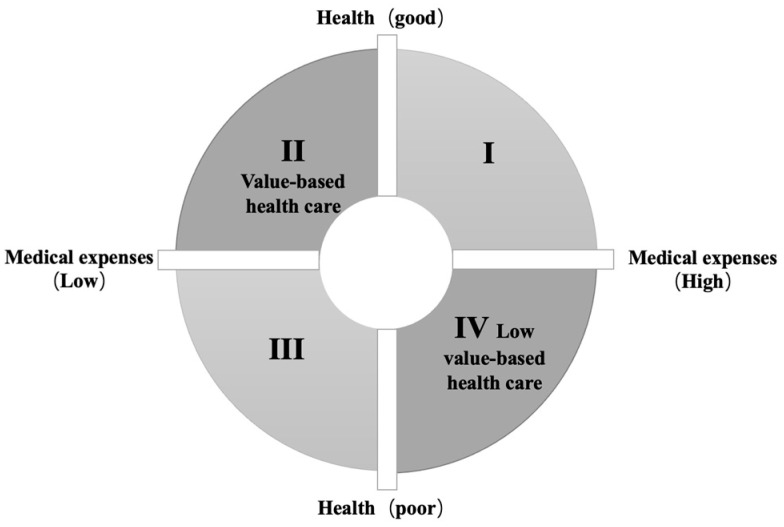
Four-dimensional figure illustrating the interactions between health and medical expenses. Note: In the second quadrant, low medical expenses and high levels of health reflect value-based health care. High medical expenses and low levels of health reflect low-value-based healthcare.

Can the challenges facing the medical care system be solved by simply controlling medical expenses? Obviously not. At the core of value-based health care is a need to balance the rational control of medical expenses sprawl against the protection of levels of health [15,17]. To illustrate this concept, we constructed a four-dimensional quadrant graph (see Figure 1) with patient health status and medical expenses as indicators. In the second quadrant, low medical expenses and high levels of health reflect value-based health care, while high medical expenses and low levels of health reflect low-value-based health care.

**Figure 2:**
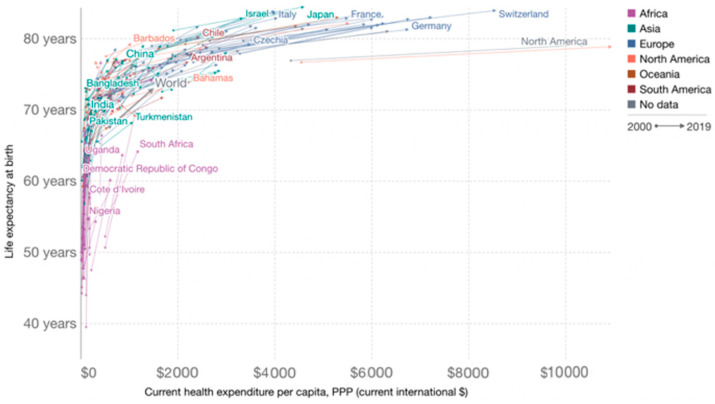
Trends in life expectancy and health expenditure by country, 2000–2019. Note: This visualization shows the cross-country relationship between life expectancy at birth (ordinate data) and healthcare expenditure per capita (abscissa). Arrows connect these two observations, showing the change over time of both measures for all countries in the world. China is at the intersection of low-value-based healthcare and value-based healthcare. Source: Image from https://ourworldindata.org/ (accessed on 20 May 2022).

Countries worldwide actively seek to match high healthcare expenditures with efficient healthcare outcomes, thus avoiding low-value-based health care. However, there are significant differences in outcomes for beneficiaries (see Figure 2). In the United States, one-third of healthcare expenditure is wasted due to neglect of care, prevention approaches [18], and low utilization of healthcare services [19]. However, European countries have actively built healthcare systems over the past decades, which have greatly improved population health [20]. The health benefits in the United States are much lower than those in European countries [21,22]. Currently, China is at the intersection of value-based health care and low-value-based health care, and its future economic development depends upon a new round of health policy reform to overcome the healthcare cost dilemma and protect the health of beneficiaries.

The contribution of this paper is two-fold: First, in a practical sense, most of the existing literature [23,24,25] focuses on the impact of LTCI on total medical expenses but ignores out-of-pocket medical expenses. Compared with total medical expenses, out-of-pocket medical expenses can better reflect the role of individuals in terms of actual expenditure [26]. Second, from a theoretical perspective, it helps enrich and expand our understanding of the LTCI microeconomic consequences from a value-based perspective. The paper focuses on the economic benefits of LTCI from the perspectives of purchase decision-making [27] and sustainability [28], and less focus is placed on whether LTCI can protect or even enhance the health of beneficiaries.

In the remainder of this paper, we review the literature on the influences of LTCI on out-of-pocket medical expenses and self-rated health and the reasons for the controversy. We then present our use of the PSM-DID method to calculate the net effect of LTCI and complete a series of robustness tests. We discuss whether LTCI can control medical expenses and protect the health of beneficiaries and conclude by presenting the contributions of our research findings and the study limitations.

## 2. Literature Review

### 2.1. Long-Term Care Insurance Policy Context

In June 2016, China’s Ministry of Human Resources and Social Security announced that 15 cities were piloting an LTCI. It was clearly stated that the LTCI should focus on meeting the costs of basic living care and medical care closely related to basic living for severely disabled people. With this as the guide, the pilot cities introduced pilot programs taking into consideration local conditions, so as to “apply policies according to the city”. Table 1 summarizes the implementation time and coverage objects of the LTCI system in the 15 pilot cities.

From the perspective of implementation time, except for Qingdao (July 2012), Changchun (May 2015), and Nantong (January 2016), the pilot cities all started the pilot after China’s Ministry of Human Resources and Social Security issued the Guidelines. There are differences in the populations covered [29]. In terms of coverage objects, Chengde City, Qiqihar City, Ningbo City, Anqing City, Shangrao City, Guangzhou City, Chongqing City, and Chengdu City were mainly covered by employee health insurance participants at the beginning of the pilot. Changchun City and Qingdao City were mainly covered by medical insurance for employees and medical insurance for non-working urban residents. Other pilot cities covered medical insurance for employees and medical insurance for urban and rural residents.

### 2.2. Long-Term Care Insurance and Medical Expenses

The relationship between LTCI and medical expenses has sparked heated discussions in the academic community, and no consensus has been reached. We can divide the extant literature into three main themes. First, LTCI has been found to reduce medical expenses. Existing studies show that LTCI can provide quality care services [30], reduce unnecessary outpatient visits [31], and shorten hospital stays [23,32] to reduce healthcare costs. For example, Gade and Venohr [30] found that providing quality care services to critically ill patients can effectively reduce the ineffective use of medical resources, reduce the number of outpatient visits, and thus reduce medical expenses. Hyun and Kang [32] found that patients receiving differing levels of care had associated reductions in the length of hospital stay: the higher the level of care, the shorter the hospital stay.

Second, there is evidence that LTCI increases medical expenses. Through the provision of essential public services to beneficiaries, they tend to acquire more knowledge [33], leading them to expect more practical support [34] and further increasing the medical burden. Likewise, because LTCI provides financial support for informal caregivers (generally family members), the family economic burden of the beneficiaries is reduced [35], which generates an “income effect” and increases medical expenditures.

Finally, there is evidence that LTCI has an insignificant effect on medical expenses. Early studies have shown that nursing facility utilization is uncorrelated with the number of hospital outpatients [36], and medical expenses partially offset the expenditures on medical care [37].

### 2.3. Long-Term Care Insurance and Health

Whether the disabled elderly can receive a reasonable level of care has an important impact on their health. However, scholars have not reached a consensus on the impact of LTCI on health. For instance, a large number of studies have shown that specialized long-term care services can improve the health status of disabled elderly people in their later years [38,39]. For example, Choi and Joung [40] investigated the mortality difference between users and non-users of socialized long-term care services in South Korea, finding that the mortality risk of disabled individuals using long-term care services was significantly lower than that of non-users. Spiers et al. [41] contend that cognitive training in LTCI can significantly improve the elderly’s daily activity ability and thus improve their health.

Moreover, owing to the differences in the accessibility of LTCI to different groups, LTCI also has uneven benefits and the potential to widen health disparities among members of society [42,43]. García-Gómez et al. [44] analyzed the data from a disabled persons survey in Spain and found that the accessibility of formal care and its high cost were the main reasons why the care needs of disabled individuals in low-income families could not be fully met. Moreover, the health effects of LTCI are actually quite small compared to factors such as genetics, environment, location, and income [45].

### 2.4. Reasons for the Controversy

There are three main reasons why the cost-lowering and health effects of LTCI remain controversial. The first is the difference in indicator measurement methods. Indicators here refer to the implementation objects of LTCI and the indicators of medical expenses. To enhance comparability and reduce selection bias, we compared LTCI pilot cities with non-pilot cities. Most scholars use total medical expenses [24,25] to measure individual medical expenses, while few scholars have focused on out-of-pocket medical expenses [46]. However, out-of-pocket medical expenses can better reflect the actual expenditure of individuals.

The second reason is the difference in model setting. Whether the relationship between LTCI and out-of-pocket medical expenses and health is linear or nonlinear is primarily affected by differences in the model set. Existing studies have adequately discussed the relationship but have not provided sufficient evidence for why the relationship is linear or nonlinear [24,47]. Empirical studies have found that LTCI for medical expenses and health can have a positive correlation, a negative correlation, a “U” shape [48,49] (a downward trend followed by an upward trend), or no correlation.

The third reason is contextual differences. Studies on the impact of LTCI on medical expenses and health are mostly concentrated in developed countries. From a macro perspective, most of the research focuses on financing models [50], market prospects [51,52], and institutional design [53]. In contrast, the micro perspective focuses on the level of care [54,55], health impact [56], and satisfaction evaluation [57,58], and studies on the micro-effects of policies in developing countries are still lacking [59,60]. Different countries have different institutional cultures and economic environments, so it is necessary to test whether the conclusions obtained from developed countries remain valid in developing countries such as China.

## 3. Materials and Methods

### 3.1. Data Source

We examined the effects of LTCI using data from four time periods (2011, 2013, 2015, and 2018) taken from the China Health and Retirement Longitudinal Study (CHARLS) database. There are three reasons for this. First, the Social Science Survey Center of Peking University uses the database under the probability proportionate to size sampling method, with middle-aged and older people aged 45 or older serving as the primary survey subjects. The database includes information on personal characteristics and medical expenses, which are pertinent to the current research topics and themes. Second, the Ministry of Human Resources and Social Security of the People’s Republic of China issued implementation opinions on “Further Promoting the Pilot Long-term Care Insurance System” in 2016, which explicitly stated that LTCI should be coordinated at the local and municipal levels. CHARLS is a high-quality micro-individual tracking survey database at the municipal level with a high level of information completeness, which can effectively reduce the bias in estimating LTCI policy effects. Third, the CHARLS database was launched in 2011 with a nationwide tracking survey, while the LTCI policy was piloted in 2016. We chose four time-sensitive data points that satisfy PSM-DID requirements. Finally, we obtained a valid sample of 35215.

### 3.2. Variable Definition and Data Description

In this research, out-of-pocket medical expenses and self-rated health were the key dependent variables. Self-rated health was measured using the question “Would you say your health is excellent, very good, good, fair, or poor?”. The measures of out-of-pocket medical expenses were as follows: out-of-pocket on inpatient costs last year, out-of-pocket on outpatient costs last month, and out-of-pocket on self-medical costs last month. Therefore, the total out-of-pocket medical expenses included out-of-pocket inpatient costs, out-of-pocket outpatient costs, and self-medical costs, of which out-of-pocket outpatient costs and self-medical costs reflect monthly data. Annualizing the monthly data did not affect the statistical analysis of causality or reduce the estimation error of out-of-pocket medical expenses. The total out-of-pocket medical expenses were processed logarithmically to ensure they were normally distributed.

The explanatory variable was the dummy variable “Treat” for whether the LTCI policy had been implemented. Since the CHARLS statistical period runs from July to September of the current year, an LTCI policy less than six months old is still subject to its provisions. For the core explanatory variable of LTCI, if the individual’s city belongs to the pilot city, it is regarded as part of the experimental group (assigned a value of 1), and otherwise, it is regarded as part of the control group (assigned a value of 0). Eleven pilot cities—Chengde City, Qiqihar City, Shanghai City, Suzhou City, Ningbo City, Anqing City, Shangrao City, Jingmen City, Guangzhou City, Chongqing City, and Chengdu City—were included in the experimental group, and all the other non-pilot cities were included in the control group. We excluded Qingdao, Chuangchun, Nantong, and Shihezi due to the extended distance from the pilot cities and missing data. We set the time variable “After” in which the cities that implemented LTCI policies in 2018 were set to 1 (after the implementation of the LTCI), and the data from 2011, 2013, and 2015 were given the value of 0 (before the implementation of the LTCI).

Control variables were defined according to the Anderson healthcare utilization model [61], in which we established an indicator system with three dimensions, consisting of predisposing, needs-based, and enabling factors, to reduce error from the omitted variables. Gender, age, marriage, and residence were used as predisposing variables. Among the needs-based variables were activities of daily living (ADL) score, Center for Epidemiological Studies-Depression (CESD) score, and chronic diseases (including blood pressure, diabetes, cancer, lung disease, heart problem, stroke, psychological problems, arthritis, dyslipidemia, liver disease, kidney disease, stomach/digestive disease, and asthma) [62]. The enabling factors included education, child, total income, and pension [63]. Table 2 displays detailed definitions of the variables.

Table 3 provides descriptive statistics for the primary variables. Among them, out-of-pocket medical expenses have a mean value of 4.5796, a standard deviation of 3.8288, a minimum value of 0, and a maximum value of 14.6220. The average self-rated health is 3.0159, with a standard deviation of 0.9463 and a range of 1 to 5. While the pattern of data for out-of-pocket medical expenses and self-rated health is comparable to that reported by Liu and Hu [64] using the same database, the data varied considerably over the sample period (e.g., total income and activities of daily living score fluctuated), confirming the need to investigate the effect of LTCI on out-of-pocket medical expenses and self-rated health.

### 3.3. Identification Strategy and Model Setting

The DID method is prone to “selectivity bias”, that is, it cannot ensure that the experimental group and control group have the same individual characteristics before the policy is implemented [65], which is quite common in the case of large samples. The samples in this paper cover several prefecture-level cities in China, and there are great regional and economic differences among the samples and obviously large individual differences. To solve this endogeneity problem and accurately measure the net effect of LTCI on out-of-pocket medical expenses and self-rated health, we used the PSM-DID method, which mainly involved two steps:

First, the Logit model was used to predict the pilot probability of LTCI, and the control variables (including retirement, age, gender, education, total income, marriage, pension, education, ADL score, CESD score, child, and residence) were used as the identification characteristics of the sample points. Cities in the control group that are close to the experimental group in the tendency score were selected as the control group [65], and the caliper radius matching method was adopted for matching. The following model is constructed by Logit regression:

$$P_{i}\left(x\right)=P_{r}\left(D_{i}=1|x_{i}\right)=Logit\left[f\left(x_{i}\right)\right]$$

where D is the dummy variable, the experimental group is 1, and the control group is 0; $f\left(x_{i}\right)$ represents a linear function of the covariables. First of all, the propensity score of out-of-pocket medical expenses and self-rated health under LTCI should be estimated by Logit regression. Secondly, we conducted the propensity score matching; the unqualified samples were eliminated after the matching results.

Second, based on the matched experimental group and control group, we used DID method to re-identify the effects of LTCI on out-of-pocket medical expenses and self-rated health. Therefore, drawing the work of related scholars [66,67], the PSM-DID regression model was as follows:

(1) $$Y_{it}^{psm}=\beta_{0}+\beta_{1}Treat_{i}\times After_{t}+\beta_{2}Treat_{i}+\beta_{3}After_{t}+\beta Control_{it}+\mu_{i}+\lambda_{t}+\varepsilon_{it}$$

where $Y_{it}^{psm}$ represents the explained variable of out-of-pocket medical expenses and self-rated health after PSM.; $Treat_{i}$ is the grouping variable, and the cities covered by the LTCI policy are 1, or 0; $After_{t}$ is the time grouping variable, whereby, 1 is 2018, and 0 is 2011, 2013, and 2015; A series of control variables such as age, gender, education, marriage, residence, activities of daily living score, child, total income, retirement, pension, Center for Epidemiological Studies-Depression score, and chronic disease make up $Control_{it}$. Specifically, the interaction term coefficient ($\beta_{1}$) of $Treat_{i}$ and $After_{t}$ is observed to estimate the treatment effect of LTCI on out-of-pocket medical expenses and self-rated health; $\varepsilon_{it}$ is a random error term.

## 4. Results

### 4.1. Time Trends in Out-of-Pocket Medical Expenses and Self-Rated Health

**Figure 3:**
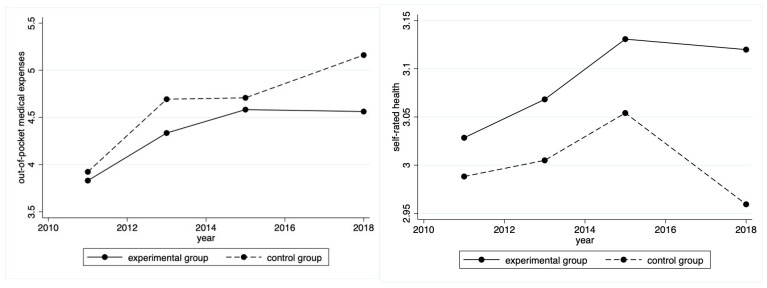
Time trends of out-of-pocket medical expenses (**left**) and self-rated health (**right**) in the experimental group and control group.

We plotted the time trends of out-of-pocket medical expenses and self-rated health between the experimental group and control group to visually reveal the difference in changes between the two groups. Figure 3 shows that before the introduction of the LTCI policy in 2016, the out-of-pocket medical expenses and self-rated health of the experimental group and control group had almost no significant changes, maintaining a basically parallel time trend. However, after the introduction of the LTCI policy, the experimental group and the control group showed a different trend of change. In terms of out-of-pocket medical expenses, the out-of-pocket medical expenses of the experimental group showed a time trend of decline, while the out-of-pocket medical expenses of the control group showed an increasing trend. In terms of self-rated health, the gap between the experimental group and control group showed a widening trend.

### 4.2. The Balance Test

The main purpose of the balance test is to check whether there are still significant differences between the covariates of the experimental group and the control group after the PSM [68]. Table 4 shows that before PSM, there were significant differences in pairing variables between the experimental group and the control group, but after PSM, there were no significant differences in pairing variables between the two groups of samples, and the absolute values of standardization deviation were all below 8% [69]. It shows that the PSM method adopted by us is effective and has passed the balance test.

### 4.3. DID Results Based on the Matched Samples

To effectively reduce the endogeneity problem, we used the PSM-DID method to confirm the impact of LTCI on out-of-pocket medical expenses and self-rated health. Table 5 shows that after accounting for the time fixed effect and individual fixed effect and adding control variables with the three dimensions of prerequisite, need, and enablement, LTCI significantly reduced the out-of-pocket medical expenses of the experimental group by 37.16% (*p* < 0.01) in the past year, and self-rated health increased by 5.73% (*p* < 0.01). These results are consistent with evidence from other countries [32,35].

### 4.4. Robustness Test

#### 4.4.1. Parallel Trend Test

**Figure 4:**
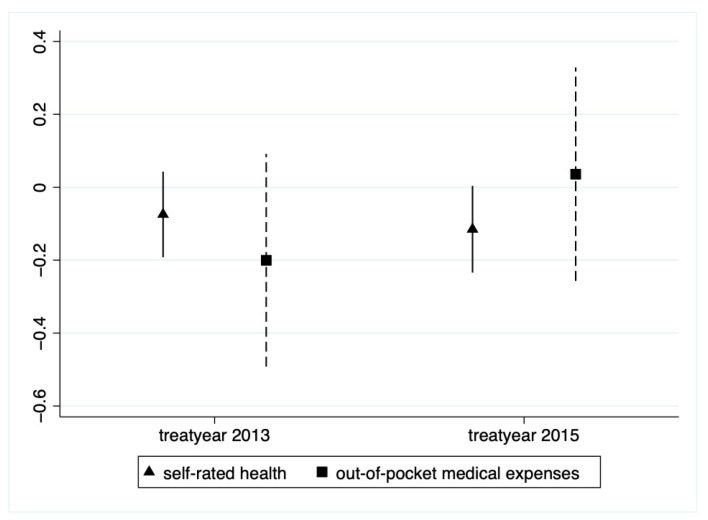
Parallel trend test.

The PSM-DID method requires that the out-of-pocket medical expenses and self-rated health of the experimental and control groups maintain basically parallel time trends before the impact of the LTCI policy. Figure 3 preliminarily verifies the parallel trend test hypothesis. Further, it is necessary to be more rigorous to ensure that this study satisfies this hypothesis. Drawing on the approach proposed by Roth [70], the treat*after, treat*year 2013, and treat*year 2015 variables were added to the regressions simultaneously (we excluded treat*year 2011 to prevent multiple co-linearities). Specifically, year2013 had a value of 1 in 2013 and 0 in all other years, and year2015 has a value of 1 in 2015 and 0 in all other years. Figure 4 shows the coefficients and 95% confidence intervals of the three periods before the introduction of LTCI. The slope of each cross-term was around 0, indicating that before the LTCI policy implementation, there was no appreciable change in the difference between the two groups.

#### 4.4.2. Placebo Effect

**Figure 5:**
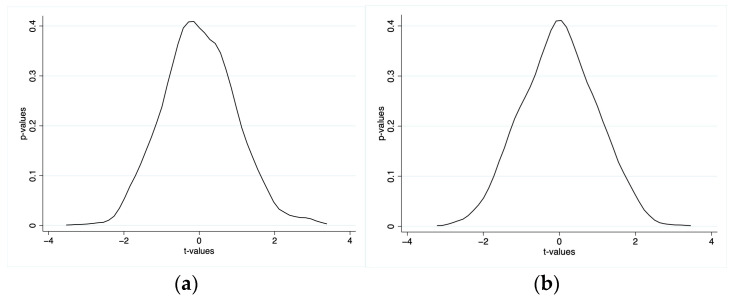
Placebo effect. (**a**) Out-of-pocket medical expenses: bandwidth = 0.2241 (Kernel: Epanechnikov); (**b**) Self-rated health: bandwidth = 0.2165 (Kernel: Epanechnikov). Note: The *X*-axis is the *t*-value, the *Y*-axis is the *p*-value, and the curve is the kernel density estimated distribution.

Testing for a potential placebo effect was necessary to rule out the effects of other exogenous events (such as healthcare reform measures like the critical illness insurance program) and confirm that the findings were the result of LTCI. Drawing on the work of relevant scholars [71,72], we took 1000 random samples to represent the two study groups, and the specific kernel density distribution results are shown in Figure 5. Most of the absolute values of the estimated coefficients of sampling “*t*-values” were within the value of 2, and the “*p*-values” were above 0.1. This outcome indicates that the impact of LTCI policies was present in these 1000 random samples. This indicates that LTCI had no significant effect in the 1000 random samples. Therefore, the effect of LTCI on out-of-pocket medical expenses and self-rated health in the pilot cities is not causally related to other unknown factors.

#### 4.4.3. Tail-Curtailing

Outliers in the data can potentially affect regression results. Therefore, we tailored the continuous variables at the 1% and 99% percentiles to lessen the effect of extreme values. After tailoring, the minimum value of out-of-pocket medical expenses was 0, the maximum value was 10.9296, the mean was 4.5735, and the standard deviation was 3.8178. Table 6 shows that the interaction term coefficient remained significant (−0.3713; 0.0573) when the model (1) was repeated for the regression, demonstrating the reliability of the findings.

#### 4.4.4. Change the PSM Matching Mode

In order to test the robustness of regression results, we changed the matching method of PSM. Based on Mahalanobis matching and nearest neighbor matching, Logit was used to estimate the tendency score, and the treatment and control groups were matched. After matching, the absolute value of the standard bias of the variables in the treatment and control groups was reduced by more than 50%, and the corresponding absolute value of common bias was less than 10% [66,67]. Meet the standard after matching.

Table 7 shows that LTCI still significantly reduced out-of-pocket medical expenses and improved self-rated health, indicating the reliability and robustness of the previous PSM-DID results.

### 4.5. Heterogeneity Test

The results showed that LTCI could significantly reduce out-of-pocket medical expenses and increase self-rated health. As LTCI policies are selective and were gradually expanded in response to regional characteristics, the impact of selective policies may vary across regions and individuals with different health conditions. Therefore, we examined cross-sectional differences in the impact of LTCI using group regressions: urban–rural heterogeneity and disabled and non-disabled heterogeneity.

#### 4.5.1. Urban and Rural Heterogeneity Analysis

Due to the long-standing urban and rural dual structure in China [73], the impact of LTCI on the disabled elderly in urban versus rural areas may be heterogeneous [71]. We conducted a PSM-DID regression analysis with a sample of regions at the time of the survey. As shown in Table 8, for rural areas, LTCI reduced out-of-pocket medical expenses and increased self-rated health but insignificantly. For urban areas, LTCI significantly decreased out-of-pocket medical expenses by 40.91% (*p* < 0.01), and self-rated health increased by 5.50% (*p* < 0.1).

In summary, LTCI significantly reduced out-of-pocket medical expenses and improved self-rated health in urban areas but was insignificant in rural areas. Since urban areas are the focus of LTCI policy, they are more impacted. In contrast, rural areas do not fully enjoy the policy dividend due to the accessibility of the policy scope. As such, individuals in these areas must avoid adverse selection and moral hazards to obtain value support and path support in the system design of LTCI policy.

#### 4.5.2. Disabled and Non-Disabled Heterogeneity Analysis

The most fundamental group in LTCI coverage is the disabled and semi-disabled population. The triple difference estimator (DDD) was selected to determine the net effect of LTCI. Expressly, drawing on Olden and Møen [74], we set the variable “Dis”, and those who were unable to complete one item on the ADL scale were defined as disabled and set to 1 and 0 otherwise. The DDD model regression model was as follows:

(2) $$Y_{it}=\beta_{0}+\beta_{1}\left(Treat_{i}\times After_{t}\times Dis_{it}\right)+\beta_{2}\left(Treat_{i}\times After_{t}\right)+\beta_{3}\left(Treat_{i}\times Dis_{it}\right)+\beta_{4}\left(After_{t}\times Dis_{it}\right)+\;\beta_{5}Dis_{it}+\beta Control_{it}+\mu_{i}+\lambda_{t}+\varepsilon_{it}$$

Table 9 shows that LTCI can significantly reduce the out-of-pocket medical expenses and improve the self-rated health of disabled people, while the effect for non-disabled people is insignificant. This is mainly because LTCI takes formal care as the main payment scope and gives priority to disabled people. At the same time, by providing a financial subsidy to beneficiaries, LTCI has the potential effect of improving the nutrition and other health inputs of the beneficiaries, leading to improvement in health. For the disabled, there is often no need to cover total medical expenses because LTCI uses a third-party payment mechanism that reduces individual costs.

## 5. A Brief Discussion on Cost-Benefit Analysis

Cost-benefit analysis can effectively reflect the proportional relationship between inputs and outputs [59]. In this paper, “cost” refers to the out-of-pocket medical expenses invested. Meanwhile, “benefit” corresponds to the benefits obtained by the beneficiaries in health effects. Currently, the aging situation in China is of critical importance [75], and the primary medical service system needs to be improved. It is predicted that without effective cost-control measures, the Chinese government’s total health expenditure will triple by 2060 [76]. A sensible approach would be to work towards Pareto optimality (an ideal state where all resources are reasonably allocated). This paper refers to the reasonable allocation of medical resources and the improvement of health provision through the effective operation of LTCI. The instrumental and constructive characteristics of LTCI also meet the requirements of the “Healthy China 2030” plan.

From an instrumental perspective, LTCI can bring economic benefits by reducing the cost of family care through financial subsidies [35], thereby reducing the economic burden of families [77,78] and improving the market participation rate and wage level of workers [79,80]. It should also be noted that the potential impact of financial subsidies on beneficiaries is to improve their nutrition and other health inputs, leading to lower utilization of medical services and lower medical costs. Furthermore, LTCI will increase the total medical expenses due to the third-party payment mechanism, and most of the literature confirms that the improvement of the health status of beneficiaries is reasonable due to the increase of total medical expenses [47,64].

From the perspective of constructiveness, LTCI can produce a knowledge effect, that is, access to more nursing and medical insurance knowledge [33], and improvement in health management awareness and risk awareness. LTCI protects and even improves the health of beneficiaries by providing high-quality formal home care services [81,82,83]. From the perspective of individuals, individuals often do not pay the total medical expenses; subject to the law of supply and demand and the decline of personal payment costs, the beneficiaries tend to receive more medical care services [16].

In conclusion, LTCI can not only effectively reduce out-of-pocket medical expenses but also effectively improve the health of caretakers to achieve value-based health care. We consider that from an economic perspective, the actual value of LTCI tends to be underestimated, making these cost–benefit analysis results more conservative.

## 6. Conclusions and Implications

LTCI is one of the crucial means by which we can actively manage the aging population and meet the care needs of disabled and semi-disabled people. This paper is based on the data of CHARLS from 2011, 2013, 2015, and 2018. The findings show that LTCI can significantly reduce out-of-pocket medical expenses and improve self-rated health of beneficiaries, which is also in line with the concept of value-based health care. Further research found that LTCI had a greater impact on urban and disabled people. The results were found to be stable in the robustness tests conducted.

The findings from this study have substantial policy and practical implications. First, in China, there are a large number of disabled and semi-disabled people in rural areas, which is the most important target group of LTCI, but one which has long been neglected. More refined rules should be explored in the future LTCI treatment design, including differentiated reimbursement rates for people with different degrees of disability and more guarantees for rural groups. Second, we should ensure that health investment does not equal increased spending on health funds. The sprawling nature of health funds often leads to excessive waste of resources and deviates from the “intensive care and neglected medicine” target. We should shift from the need for medical treatment to the need for care.

## 7. Limitations and Future Directions

There are several limitations to this paper. Due to the short duration of the LTCI pilot and the small sample size of the treatment group, we used a series of robustness measures such as propensity score matching to enhance confidence in the results. Likewise, information in the questionnaire on out-of-pocket medical expenses and self-rated health was self-reported by the respondents and is prone to recall bias in people aged over 60 years. There are differences in the mode of providing care services for the disabled elderly in different cities in China [84,85], and the research on value-based health care involves multiple specialties and disciplines, being a typically complex system. Limited by the lack of comprehensive academic level and integration, this paper does not analyze the benefits of value-based health care without nursing mode, and this will be developed in the follow-up research.

## Figures and Tables

**Table 1:** Implementation time and coverage objects of the LTCI pilot program in 15 pilot cities.

Pilot City	Implementation Time	Coverage Objects
Chengde City Hebei province	November 2016	Employee health insurance participants
Changchun City Jilin province	May 2015	Medical insurance for employees and medical insurance for non-working urban residents
Qiqihar City Heilongjiang province	October 2017	Employee health insurance participants
Shanghai City	January 2017	Medical insurance for employees and medical insurance for urban and rural residents
Nantong City Jiangsu province	January 2016	Medical insurance for employees and medical insurance for urban and rural residents
Suzhou City Jiangsu province	June 2017	Medical insurance for employees and medical insurance for urban and rural residents
Ningbo City Zhejiang province	December 2017	Employee health insurance participants
Anqing City Anhui province	January 2017	Employee health insurance participants
Shangrao City Jiangxi province	January 2017	Employee health insurance participants
Qindao City Shandong province	July 2012	Medical insurance for employees and medical insurance for non-working urban residents
Jingmen City Hubei province	November 2016	Medical insurance for employees and medical insurance for urban and rural residents
Guangzhou City Guangdong province	August 2017	Employee health insurance participants
Chongqing City	December 2017	Employee health insurance participants
Chengdu City Sichuan province	July 2017	Employee health insurance participants
Shihezi City Xinjiang Production and Construction Corps	January 2017	Medical insurance for employees and medical insurance for urban and rural residents

**Table 2:** Definitions of variables.

Variables	Definition
Explained variable	
Out-of-pocket medical expenses	Past year out-of-pocket medical expenses were logarithmized
Self-rated health	Poor = 1; fair = 2; good = 3; very good = 4; excellent = 5
Main explanatory variables	
After	After 2016 = 1; others = 0
Treat	Cities covered by long-term care insurance = 1; others = 0
Control variable	
Age	Age of the elderly
Gender	Female= 1; male= 0
Residence	Rural = 1; urban = 0
Married	Living with a spouse = 1; others = 0
Education	Uneducated = 0; primary school = 6; junior high school = 9; high school and technical secondary school = 12; junior college = 15; undergraduate = 16; Master’s degree or above = 19
Chronic disease	Having a chronic disease = 1; others = 0
Activities of daily living score	Scores range from 0 to 6, with higher scores indicating poorer health
Center for Epidemiological Studies-Depression score	Scores range from 0 to 30, with higher scores indicating a higher level of depression
Retirement	Formal retirement = 1; others = 0
Child	Number of living children
Total income	Total household income was logarithmized
Pension	Have a pension = 1; others = 0

Note: Data source: CHARLS.

**Table 3:** Descriptive statistics for the main study variables.

Variables	Number	Mean	Standard Deviation	Minimum	Maximum
Out-of-pocket medical expenses	35,215	4.5796	3.8288	0	14.6220
Self-rated health	35,215	3.0159	0.9463	1	5
Age	35,215	60.8659	8.8558	45	108
Gender	35,215	0.4715	0.4992	0	1
Education	35,215	4.7524	4.6087	0	19
Marriage	35,215	0.8372	0.3692	0	1
Residence	35,215	0.6448	0.4786	0	1
Activities of daily living score	35,215	0.3459	0.9232	0	6
Child	35,215	2.6535	1.2797	0	10
Total income	35,215	9.4615	2.2245	0	14.8589
Retirement	35,215	0.1346	0.3413	0	1
Pension	35,215	0.3434	0.4749	0	1
Center for Epidemiological Studies-Depression score	35,215	8.1108	6.1135	0	30
Chronic disease	35,215	0.7636	0.4249	0	1

**Table 4:** The balance test.

Variables	Unmatched	Mean		%Bias	%Reduct Bias	*t*-Test	
	Matched	Treated	Control			t	p > t
Age	U	61.294	60.732	6.4	80.1	4.02	0.000
	M	61.294	61.405	−1.3		−0.62	0.537
Gender	U	0.470	0.471	−0.3	−305.1	−0.17	0.869
	M	0.470	0.465	1.1		0.51	0.610
Education	U	4.673	4.750	−1.7	94.4	−1.06	0.291
	M	4.673	4.669	0.1		0.04	0.965
Marriage	U	0.852	0.836	4.2	90.3	2.63	0.008
	M	0.852	0.850	0.4		0.20	0.840
Residence	U	0.565	0.661	−19.8	94.5	−12.71	0.000
	M	0.565	0.560	1.1		0.51	0.608
ADL score	U	0.298	0.348	−5.5	88.7	−3.43	0.001
	M	0.298	0.304	−0.6		−0.31	0.754
Child	U	2.439	2.671	−18.1	93.6	−11.72	0.000
	M	2.439	2.454	−1.2		−0.56	0.575
Total income	U	9.702	9.436	12.2	93.6	7.65	0.000
	M	9.702	9.685	0.8		0.39	0.697
Retirement	U	0.186	0.121	18.2	95.1	12.30	0.000
	M	0.186	0.189	−0.9		−0.40	0.692
Pension	U	0.355	0.342	2.8	71.7	1.77	0.077
	M	0.355	0.359	−0.8		−0.38	0.706
CESD score	U	7.391	8.173	−12.9	96.4	−8.17	0.000
	M	7.391	7.419	−0.5		−0.23	0.819
Chronic diseases	U	0.763	0.762	0.1	−359.7	0.08	0.938
	M	0.763	0.765	−0.6		−0.27	0.784

**Table 5:** DID results with matched samples.

Variables	Out-of-Pocket Medical Expenses		Self-Rated Health	
Treat *After	−0.4031 *** (0.0007)	−0.3716 *** (0.0017)	0.0660 ** (0.0128)	0.0573 *** (0.0281)
Age		0.0227 (0.8538)		−0.0444 (0.1007)
Marriage		−0.0102 (0.9169)		−0.0198 (0.3539)
ADL score		0.2882 *** (0.0000)		−0.0883 *** (0.0000)
Child		0.1545 *** (0.0024)		0.0071 (0.5246)
Total income		0.0265 ** (0.0114)		0.0036 (0.1141)
Retirement		−0.1245 (0.3894)		0.0417 (0.1900)
Pension		0.0497 (0.3814)		0.0137 (0.2713)
CESD score		0.0614 *** (0.0000)		−0.0228 *** (0.0000)
Chronic diseases		0.6881 *** (0.0000)		−0.1420 *** (0.0000)
Constant	3.8997 *** (0.0000)	0.9217 (0.8965)	3.0004 *** (0.0000)	5.8189 *** (0.0002)
Time fixed effect	YES	YES	YES	YES
Individual fixed effect	YES	YES	YES	YES
Number	35215	35215	35215	35215
R-squared	0.0237	0.0395	0.0035	0.0375

Note: *p*-values in parentheses, * *p* < 0.1, ** *p* < 0.05, *** *p* < 0.01. Data source: calculated by the authors.

**Table 6:** Tail-curtailing.

Variables	Out-of-Pocket Medical Expenses		Self-Rated Health	
Treat *After	−0.4028 *** (0.0007)	−0.3713 *** (0.0017)	0.0660 ** (0.0128)	0.0573 ** (0.0280)
Age		0.0247 (0.8400)		−0.0444 (0.1007)
Marriage		−0.0102 (0.9158)		−0.0198 (0.3543)
ADL score		0.2856 *** (0.0000)		−0.0883 *** (0.0000)
Child		0.1549 *** (0.0023)		0.0071 (0.5250)
Total income		0.0271 *** (0.0096)		0.0037 (0.1056)
Retirement		−0.1229 (0.3940)		0.0416 (0.1904)
Pension		0.0505 (0.3720)		0.0137 (0.2732)
CESD score		0.0608 *** (0.0000)		−0.0228 *** (0.0000)
Chronic diseases		0.6880 *** (0.0000)		−0.1420 *** (0.0000)
Constant	3.8971 *** (0.0000)	0.7982 (0.9100)	3.0004 *** (0.0000)	5.8182 *** (0.0002)
Time fixed effect	YES	YES	YES	YES
Individual fixed effect	YES	YES	YES	YES
Number	35215	35215	35215	35215
R-squared	0.0236	0.0393	0.0035	0.0375

Note: *p*-values in parentheses, * *p* < 0.1, ** *p* < 0.05, *** *p* < 0.01. Data source: calculated by the authors.

**Table 7:** Change the PSM matching mode.

Variables	Mahalanobis Distance Matching		Nearest Neighbor Matching	
	Out-of-Pocket Medical Expenses	Self-Rated Health	Out-of-Pocket Medical Expenses	Self-Rated Health
Treat *After	−0.3716 *** (0.0017)	0.0573 ** (0.0281)	−0.3716 *** (0.0017)	0.0576 ** (0.0272)
Age	0.0227 (0.8538)	−0.0444 (0.1007)	0.0227 (0.8539)	−0.0436 (0.1074)
Marriage	−0.0102 (0.9169)	−0.0198 (0.3539)	−0.0102 (0.9169)	−0.0198 (0.3541)
ADL score	0.2882 *** (0.0000)	−0.0883 *** (0.0000)	0.2883 *** (0.0000)	−0.0884 *** (0.0000)
Child	0.1545 *** (0.0024)	0.0071 (0.5246)	0.1545 *** (0.0024)	0.0071 (0.5248)
Total income	0.0265 ** (0.0114)	0.0036 (0.1141)	0.0265 ** (0.0114)	0.0036 (0.1134)
Retirement	−0.1245 (0.3894)	0.0417 (0.1900)	−0.1245 (0.3894)	0.0416 (0.1904)
Pension	0.0497 (0.3814)	0.0137 (0.2713)	0.0496 (0.3825)	0.0137 (0.2715)
CESD score	0.0614 *** (0.0000)	−0.0228 *** (0.0000)	0.0614 *** (0.0000)	−0.0228 *** (0.0000)
Chronic diseases	0.6881 *** (0.0000)	−0.1420 *** (0.0000)	0.6881 *** (0.0000)	−0.1421 *** (0.0000)
Constant	0.9217 (0.8965)	5.8189 *** (0.0002)	0.9221 (0.8965)	5.7715 *** (0.0002)
Time fixed effect	YES	YES	YES	YES
Individual fixed effect	YES	YES	YES	YES
Number	35215	35215	35213	35213
R-squared	0.0395	0.0375	0.0395	0.0375

Note: *p*-values in parentheses, * *p* < 0.1, ** *p* < 0.05, *** *p* < 0.01. Data source: calculated by the authors.

**Table 8:** Urban and rural heterogeneity analysis.

Variables	Out-of-Pocket Medical Expenses		Self-Rated Health	
	Urban	Rural	Urban	Rural
Treat *After	−0.4091 *** (0.0027)	−0.2427 (0.3778)	0.0550 * (0.0738)	0.0430 (0.4230)
Age	−0.0731 (0.5951)	0.4680 (0.1200)	−0.0416 (0.1803)	−0.0703 (0.2310)
Marriage	−0.0324 (0.7642)	−0.0378 (0.8870)	0.0119 (0.6260)	−0.2023 *** (0.0001)
ADL score	0.2915 *** (0.0000)	0.1698 * (0.0609)	−0.0854 *** (0.0000)	−0.0843 *** (0.0000)
Child	0.1606 *** (0.0047)	0.2184 (0.1062)	0.0056 (0.6613)	0.0214 (0.4166)
Total income	0.0290 ** (0.0118)	0.0158 (0.6118)	0.0047 * (0.0698)	0.0048 (0.4289)
Retirement	−0.1668 (0.4755)	0.0042 (0.9847)	0.0461 (0.3825)	0.0486 (0.2506)
Pension	0.0363 (0.5799)	0.2186 (0.1621)	0.0125 (0.3981)	0.0353 (0.2477)
CESD score	0.0583 *** (0.0000)	0.0781 *** (0.0000)	−0.0220 *** (0.0000)	−0.0300 *** (0.0000)
Chronic diseases	0.6670 *** (0.0000)	0.7580 *** (0.0008)	−0.1406 *** (0.0000)	−0.1260 *** (0.0041)
Constant	6.3403 (0.4217)	−25.0523 (0.1563)	5.5914 *** (0.0017)	7.5903 ** (0.0277)
Time fixed effect	YES	YES	YES	YES
Individual fixed effect	YES	YES	YES	YES
Number	27894	6320	27894	6320
R-squared	0.0414	0.0349	0.0359	0.0513

Note: *p*-values in parentheses, * *p* < 0.1, ** *p* < 0.05, *** *p* < 0.01; Data source: calculated by the authors.

**Table 9:** Disabled and non-disabled heterogeneity analysis.

Variables	Out-of-Pocket Medical Expenses		Self-Rated Health	
	Non-Disabled	Disabled	Non-Disabled	Disabled
Treat *After * Dis	−0.4588 (0.2135)	−0.4002 *** (0.0031)	−0.0726 (0.4074)	0.0685 ** (0.0191)
Age	0.2978 (0.4255)	−0.0828 (0.5593)	−0.0520 (0.5582)	−0.0204 (0.5057)
Marriage	−0.3799 (0.2290)	0.0592 (0.5976)	−0.0450 (0.5488)	−0.0234 (0.3346)
Child	0.1971 (0.1977)	0.1262 ** (0.0321)	−0.0380 (0.2955)	0.0108 (0.3951)
Total income	0.0279 (0.3775)	0.0265 ** (0.0286)	0.0047 (0.5284)	0.0034 (0.1967)
Retirement	1.2741 ** (0.0353)	−0.1856 (0.2389)	−0.0687 (0.6327)	0.0366 (0.2830)
Pension	0.1623 (0.3068)	0.0408 (0.5428)	0.0103 (0.7848)	0.0201 (0.1656)
CESD score	0.0500 *** (0.0000)	0.0651 *** (0.0000)	−0.0213 *** (0.0000)	−0.0230 *** (0.0000)
Chronic diseases	0.2945 (0.4229)	0.6427 *** (0.0000)	0.0456 (0.6013)	−0.1479 *** (0.0000)
Constant	−14.0457 (0.5370)	6.8173 (0.3984)	5.9572 (0.2704)	4.4901 ** (0.0101)
Time fixed effect	YES	YES	YES	YES
Individual fixed effect	YES	YES	YES	YES
Number	28959	6256	28959	6256
R-squared	0.0275	0.0324	0.0412	0.0245

Note: *p*-values in parentheses, * *p* < 0.1, ** *p* < 0.05, *** *p* < 0.01. Data source: calculated by the authors.

## Data Availability

Publicly available datasets were analyzed in this study. This data can be found here: https://charls.charlsdata.com/pages/Data/2011-charls-wave1/zh-cn.html, (accessed on 20 May 2022). https://charls.charlsdata.com/pages/Data/2013-charls-wave2/zh-cn.html, (accessed on 20 May 2022). https://charls.charlsdata.com/pages/Data/2015-charls-wave4/zh-cn.html, (accessed on 20 May 2022). https://charls.charlsdata.com/pages/Data/2018-charls-wave4/zh-cn.html, (accessed on 20 May 2022). https://charls.charlsdata.com/pages/Data/harmonized_charls/zh-cn.html (accessed on 20 May 2022).
